# Supplementary material for: Bat power-metabolic profiling of the Egyptian fruit bat Rousettus aegyptiacus reveals distinctive cardiac adaptations
Source: bioRxiv. 2025 Nov 16:2025.04.16.649087. Preprint. [Version 2] doi: 10.1101/2025.04.16.649087 (PMC12642545; doi:10.1101/2025.04.16.649087)
Supplement: 1 [file NIHPP2025.04.16.649087V2-supplement-1.pdf]

bioRxiv preprint doi: https://doi.org/10.1101/2025.04.16.649087; this version posted November 16, 2025. The copyright holder for this preprint (which was not certified by peer review) is the author/funder, who has granted bioRxiv a license to display the preprint in perpetuity. It is made available under aCC-BY 4.0 International license.

Supplementary Figures

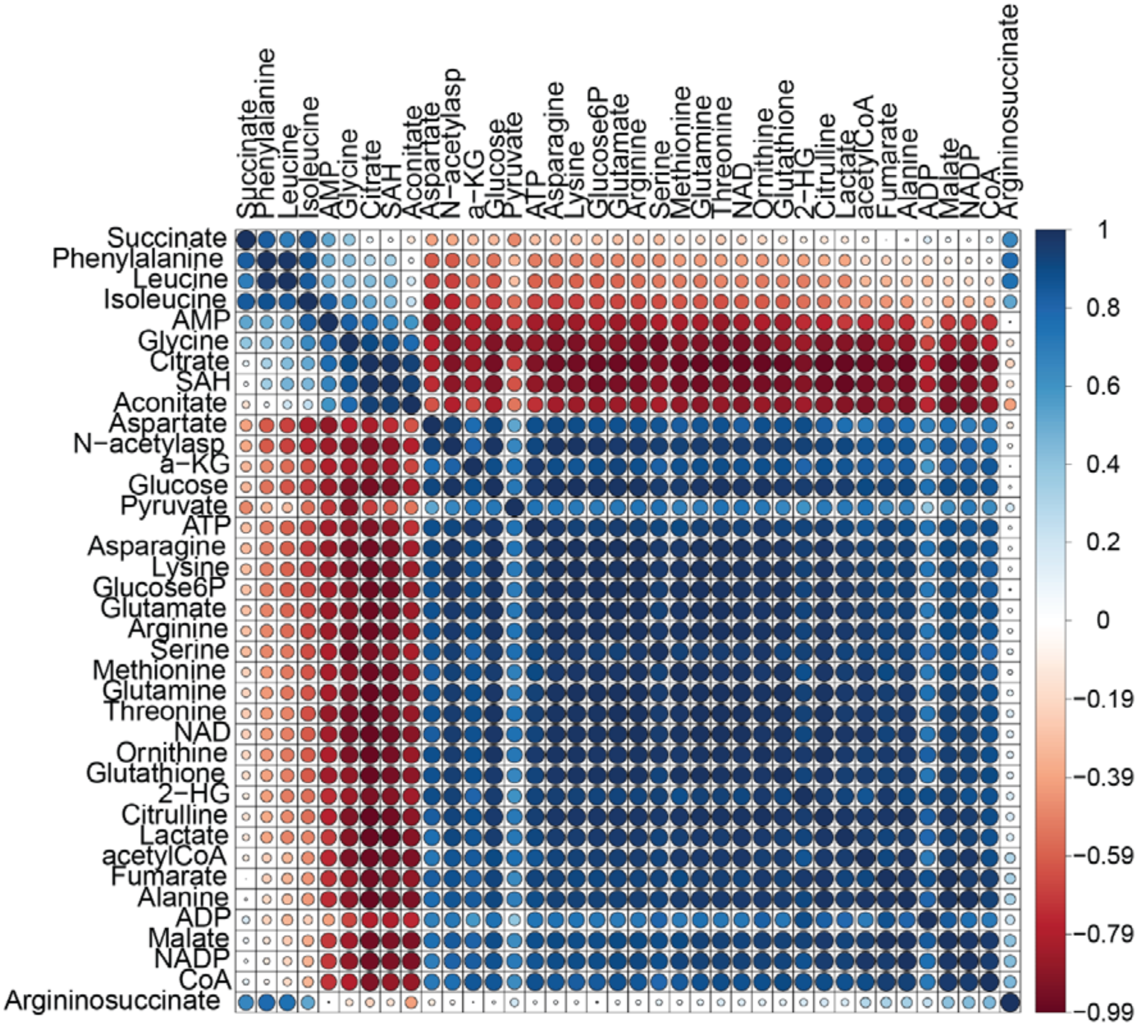

Supplementary Figure 1. Correlation analysis of bat metabolome. Metabolite abundances from NMR- and LC-MS/MS-based metabolomics were analysed using Spearman correlation to reveal interactions between intermediates. Positively and negatively correlating intermediates are indicated by blue and red colour coding, respectively. Circle sizes indicate calculated absolute spearman coefficient ranging from 0 to 1.

bioRxiv preprint doi: https://doi.org/10.1101/2025.04.16.649087; this version posted November 16, 2025. The copyright holder for this preprint (which was not certified by peer review) is the author/funder, who has granted bioRxiv a license to display the preprint in perpetuity. It is made available under aCC-BY 4.0 International license.

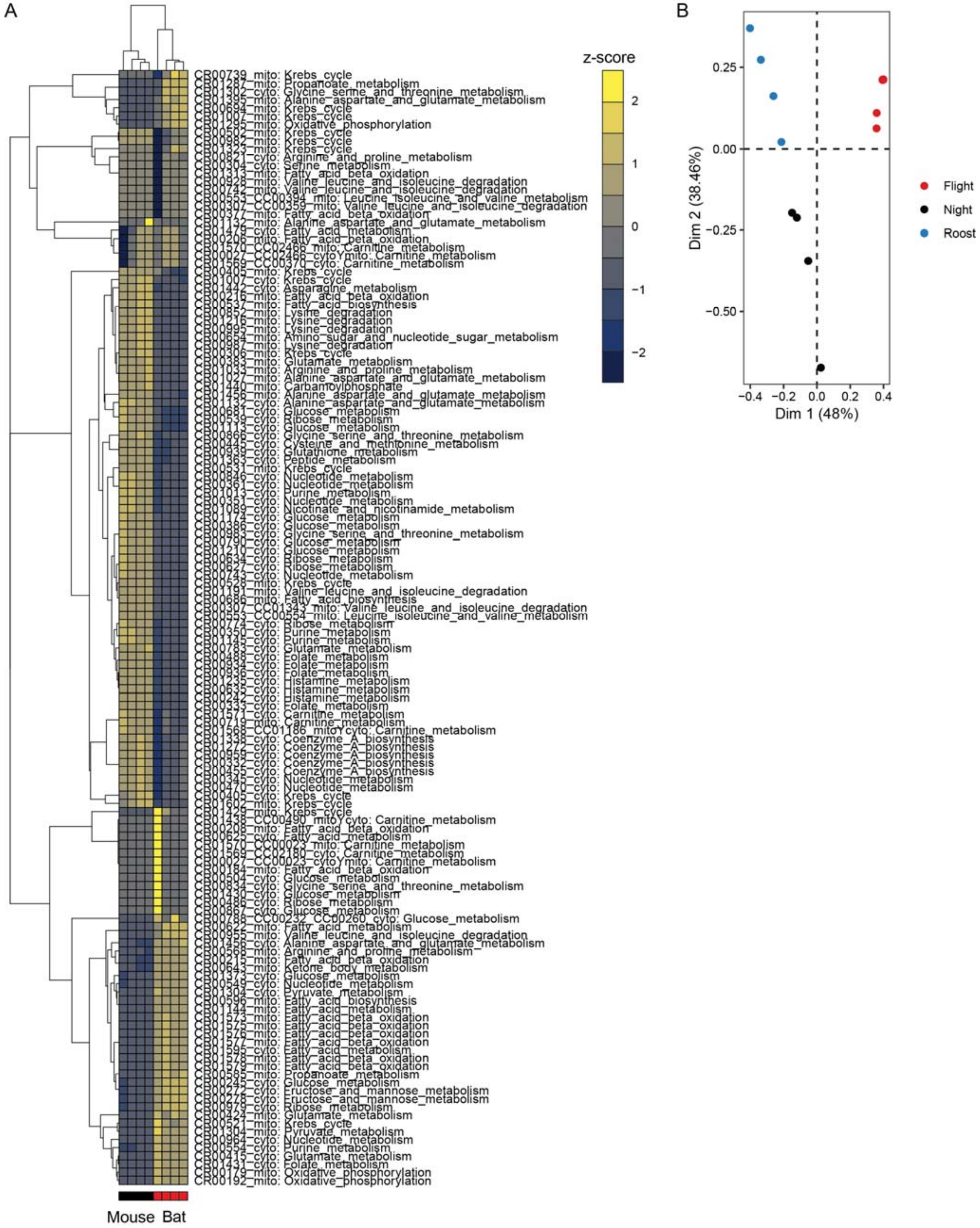

Supplementary Figure 2. *In silico* modelling of bat metabolism. (**A**) Unsupervised hierarchical cluster analysis and heatmap of significantly altered metabolic fluxes in bat and mouse simulations using CardioNet. Flux rates were z-score normalised. n=4/species. (**B**) Principal component analysis of simulated activity states: flight, night and roost.
